# Development and validation of reversed-phase HPLC gradient method for the estimation of efavirenz in plasma

**DOI:** 10.1371/journal.pone.0174777

**Published:** 2017-05-15

**Authors:** Shweta Gupta, Rajesh Kesarla, Narendra Chotai, Abdelwahab Omri

**Affiliations:** 1 Department of Pharmaceutical Technology, Parul University, Vadodara, Gujarat, India; 2 Department of Pharmaceutics, ISF College of Pharmacy, Moga, Punjab, India; 3 Department of Pharmaceutics, A. R. College of Pharmacy, Anand, Gujarat, India; 4 Department of Chemistry& Biochemistry, Laurentian University, Sudbury, Ontario, Canada; Helsingin Yliopisto, FINLAND

## Abstract

Efavirenz is an anti-viral agent of non-nucleoside reverse transcriptase inhibitor category used as a part of highly active retroviral therapy for the treatment of infections of human immune deficiency virus type-1. A simple, sensitive and rapid reversed—phase high performance liquid chromatographic gradient method was developed and validated for the determination of efavirenz in plasma. The method was developed with high performance liquid chromatography using Waters X-Terra Shield, RP18 50 x 4.6 mm, 3.5 μm column and a mobile phase consisting of phosphate buffer pH 3.5 and Acetonitrile. The elute was monitored with the UV-Visible detector at 260 nm with a flow rate of 1.5 mL/min. Tenofovir disoproxil fumarate was used as internal standard. The method was validated for linearity, precision, accuracy, specificity, robustness and data obtained were statistically analyzed. Calibration curve was found to be linear over the concentration range of 1–300 μg/mL. The retention times of efavirenz and tenofovir disoproxil fumarate (internal standard) were 5.941 min and 4.356 min respectively. The regression coefficient value was found to be 0.999. The limit of detection and the limit of quantification obtained were 0.03 and 0.1 μg/mL respectively. The developed HPLC method can be useful for quantitative pharmacokinetic parameters determination of efavirenz in plasma.

## Introduction

**Fig 1:**
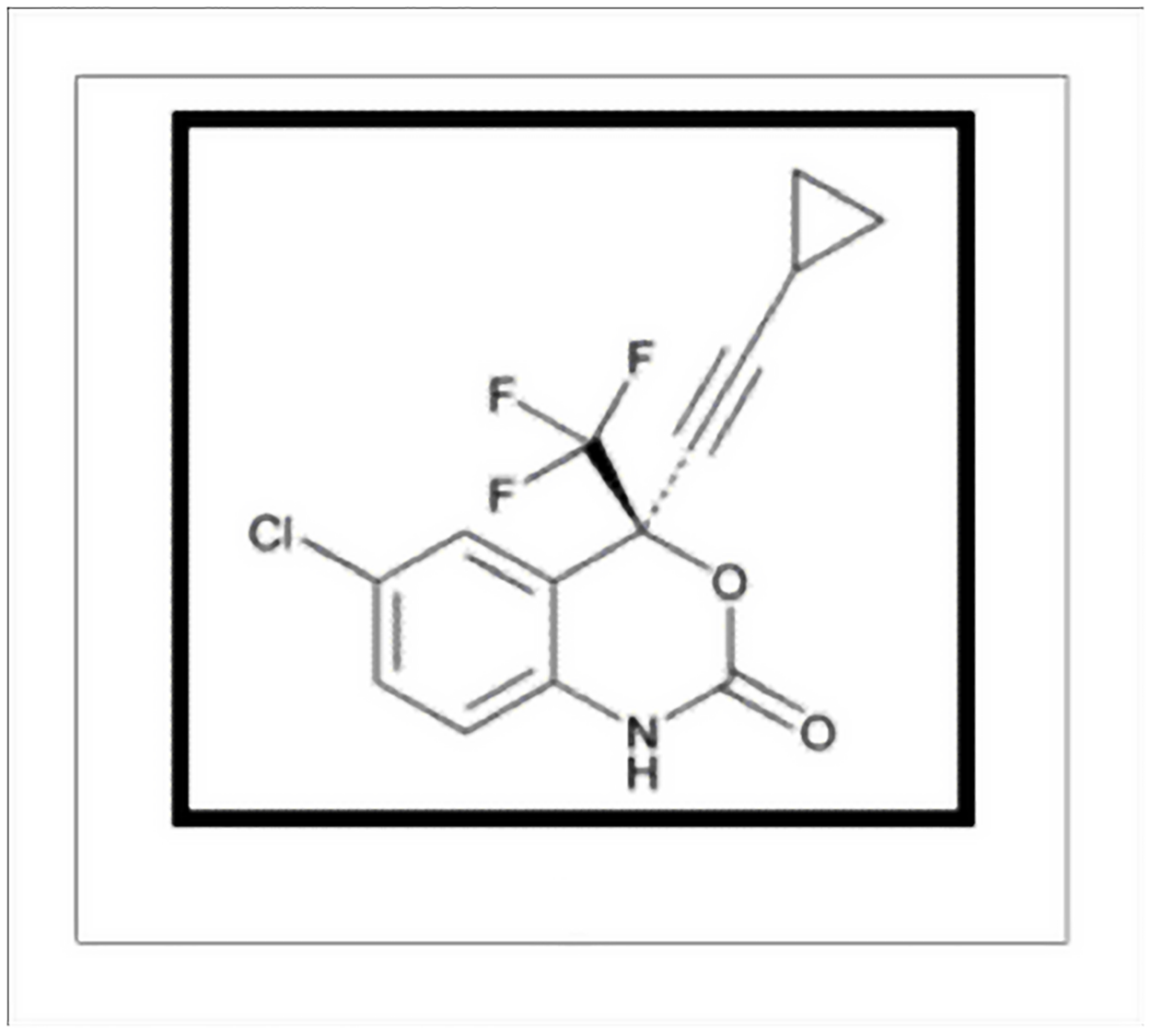
Structure of efavirenz.

Efavirenz (EFV) is a non-nucleoside reverse transcriptase inhibitor (NNRTI) and is used as part of highly active antiretroviral therapy (HAART) for the treatment of infections of human immunodeficiency virus (HIV) type 1 causing Acquired Immuno-Deficiency Syndrome (AIDS). EFV is one of the preferred NNRTI for treating HIV infection, particularly for those HIV infections which previously has not been treated [1]. The current recommended first line of treatment is a NNRTI, EFV [2]. EFV is also used in combination with other antiretroviral agents as part of an expanded post-exposure prophylaxis regimen to prevent HIV transmission for those exposed to materials associated with a high risk for HIV transmission [2]. Chemically, EFV (C_14_H_9_ClF_3_NO_2_) is (S)-6-chloro-4-(cyclopropylethynyl)-1,4-dihydro-4-(triflouoromethyl)-2H-3,1-benzoxazin -2-one, having the structure as shown in Fig 1 [1,3].

There are a few methods reported for the estimation of EFV in bulk drug [4], pharmaceutical dosage forms [4] and plasma [5–7] using HPLC, HPTLC [8] and FTIR [9]. Resolution and sensitivity is reported to be higher with HPLC in comparison to HPTLC. FTIR instruments have a single beam and hence for highly sensitive work and experiments which take a long time, changes in infrared absorbing gas concentrations due to atmospheric conditions can severely affect the results. Most of the methods with HPLC have used a 250 mm column, where the run time is expected to be higher than by using a 150 mm column. The objective of the present investigation was to develop an alternative, rapid, sensitive and reliable reversed-phase HPLC gradient method for the estimation of EFV in plasma with commonly available chemical reagents and internal standard. In order to ensure the reliability, reproducibility and sensitivity of the method, the developed analytical method was validated and statistically analyzed rigorously for various parameters in accordance with ICH guidelines.

## Materials and methods

### Reagents and chemicals

EFV and Tenofovir Disoproxil Fumarate (TDF) were the generous gifts from M/s Sun Pharma Ltd. Sikkim, India and Paradise Healthcare, Nandeshari, Vadodara, Gujarat, India respectively. HPLC grade acetonitrile, methanol, water and potassium phosphate monobasic (AR grade) were procured from Merck, Mumbai, India. Ortho-phosphoric acid and ethyl acetate were obtained from Spectrochem Pvt. Ltd., Mumbai, India. All other chemicals and solvents used were of analytical grade. Prior to use, mobile phase was filtered through a 0.45 μm membrane filter (Millipore, Bedford, MA, USA). Pooled human plasma from 20 people was obtained from Parul Sevashram Hospital, Vadodara, Gujarat, India with due certification of approval from the hospital on ethical considerations (PSH/5/11/15/01).

### Instruments and chromatographic conditions

Separation and detection was carried out on gradient HPLC system (Alliance, Waters) equipped with UV detector. A C18 reverse-phase HPLC column (Waters X-Terra Shield, 50 x 4.6 mm, 3.5 μm) was utilized with phosphate buffer pH 3.5 (adjusted with orthophosphoric acid) and acetonitrile as mobile phase A and B respectively under a gradient program. The UV wavelength, flow rate and injection volume were 260 nm, 1.5 mL/min and 20 μL respectively.

### Preparation of standard solution

Stock solution of EFV (0.5 mg/mL) and TDF (1 mg/mL) were prepared by dissolving 50 mg EFV and 100 mg of TDF respectively in 100 mL diluent (methanol: water 50:50, v/v). Further dilution of 5 mL of stock solution to 100 ml with diluent was done to produce 25 ppm EFV solution and 50 ppm TDF solution for trial batches. The working standards of EFV in concentrations ranging from 1–300 μg/mL were prepared by spiking appropriate amount of the standard solution in drug-free plasma.

### Sample preparation

The plasma sample of 250μL was transferred to tubes in which 100μL of TDF (internal standard) and 50μL of EFV were added, followed by 1 mL of ethyl acetate. The samples were vortexed for 1 min and then centrifuged using REMI centrifuge (BL– 135 R) at 10000 RPM (4°C) and organic phase was evaporated via nitrogen purging. The solid residue was reconstituted with 100μL of mobile phase and 20 μL was injected into the HPLC column.

### Analytical method development

Various trials were taken by varying the column, mobile phase, pH of mobile phase, flow rate, etc. to optimize the analytical method to give good peaks with suitable retention time. Different columns like Inertsil ODS, 150X4.6mm (5.0μm) and Waters symmetry shield C18, 150X4.6mm (5.0μm), different mobile phase like 10 mM ammonium acetate with acetonitrile at different ratios, 10 mM phosphate buffer with acetonitrile at different ratios and different pH, different flow rates (1.0 and 1.5 mL/min) were tested to optimize the chromatographic conditions.

### Analytical method validation

#### Linearity and range

The linearity was established in the range of 1 to 300 μg/mL concentrations (1, 5, 10, 20, 25, 50, 100, 200, 300 μg/mL) of EFV. The concentration of TDF (internal standard) was 50 μg/mL. All the experiments were replicated thrice. The linear regression analysis of the area ratios (analyte/internal standard) vs. concentration curve was determined. The linearity was verified using estimates of correlation coefficient.

#### Precision

The precision (repeatability) of the analytical method was determined by assaying six samples at 100% test concentration and estimating standard deviation (SD) and relative standard deviation (RSD). The intra-day and inter-day precisions were also determined by analyzing three samples at three different times of the same day and on three consecutive days respectively and determining SD, RSD and analyzing the data statistically using ANOVA.

#### Accuracy

Accuracy was assessed at three concentration levels. Six replicates were analyzed at 100% concentration level and three replicates each at 50% and 150% concentrations. Assessment of accuracy was accomplished by evaluating the percent recovery of the analyte.

#### Limit of detection (LOD) and limit of quantification (LOQ)

LOD: Signals for known low concentration (0.03 μg/ml) of analyte was compared with blank sample. Signal to noise ratio was found and compared with acceptable value (2:1) [10].

LOQ: Determination of the signal to noise ratio was performed by comparing measured signals from samples with known low concentrations (six replicates of 0.1 μg/mL) of analyte with those of blank samples and was compared with typically accepted signal-to–noise ratio of 10:1 [10.]

#### Specificity

The specificity was assessed by analyzing the analyte in the presence of placebo and observing the retention times of EFV and TDF. The results were based on three replicate analyses.

#### Robustness

The robustness of the analytical procedure was measured from its capacity to remain unaffected by small, but deliberate variations in method parameters—flow rate (± 0.2 mL/min) wavelength (±2 nm), pH of mobile phase (± 0.2) to provide an indication of its reliability during normal usage.

#### System suitability test

For system suitability six replicates of drug samples were run at 25 μg/mL and 50μg/mL concentration of EFV and TDF respectively. Repeatability with respect to peak height and peak area in six replicates, peaks symmetry (tailing factor), resolution between the peaks of drugs, theoretical plates of the column (column efficiency) and retention time were determined.

#### Stability of analytical solution

Stability of the analytical solution when stored at ambient temperature was tested at 2, 4, 6, 12, 18, 24 hours and after 10 days when stored at 4°C and compared with chromatograms of freshly prepared samples. Experiments were replicated three times. Stability sample results should be within 15% of nominal concentrations. Stability was also assessed for three freeze-thaw cycles. The samples were stored at the intended storage temperature of 4°C for 24 h and then thawed unassisted at ambient temperature. When completely thawed the samples were frozen for 12–24 h under same conditions. The freeze–thaw cycle was repeated thrice and the solutions were then analyzed.

#### Statistical analysis

The data obtained were statistically evaluated with the help of Microsoft Excel. Linearity was assessed by determination of SD, correlation coefficient and linear regression equation. While accuracy of the developed analytical method was assessed by % recovery method, SD and RSD were determined for confirming the precision, robustness, etc. Intraday and interday precision was statistically analyzed using ANOVA from Analysis Toolpak of Microsoft Excel. Signal-to-noise ratios were determined for LOD and LOQ.

## Results and discussion

### Method development and optimization

**Fig 2:**
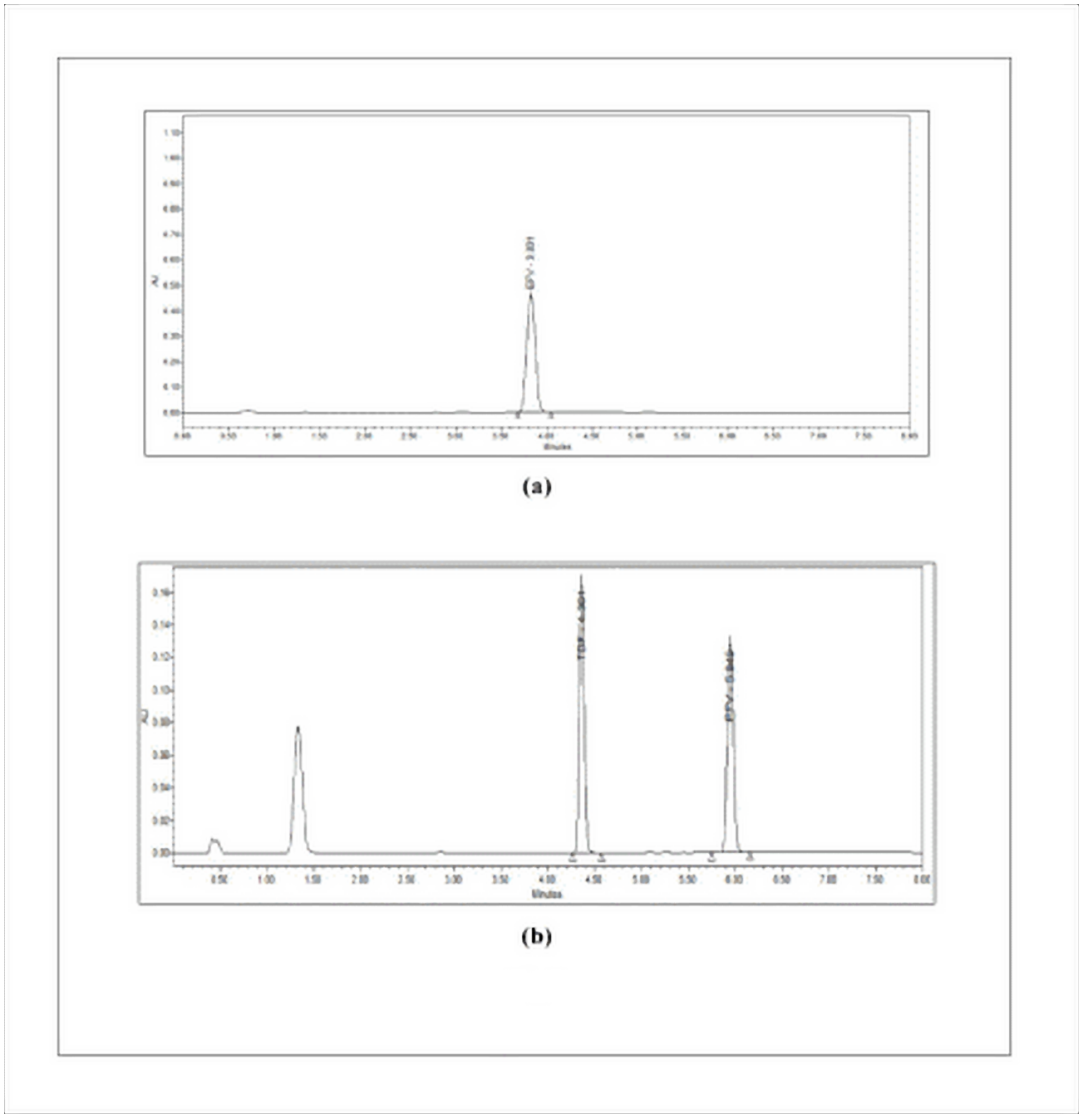
Representative chromatographic peaks obtained during analytical method development.

Several trials were taken with various columns, mobile phase, pH of the mobile phase to select the chromatographic conditions giving good peak characteristics. Among the different columns, Waters symmetry shield C18 (150 x 4.6 mm, 5.0μm) analytical column and mobile phase consisting of phosphate buffer (pH 3.5) and acetonitrile (30:70), the flow rate of 1.5mL/min was found to give good peak symmetry for EFV as shown in Fig 2(a).

In the presence of TDF as internal standard, the peak characteristics were good but the resolution between both the peaks was not appropriate, the difference in the retention time for EFV and TDF were observed to be very close. With a few modifications in the chromatographic conditions (column changed to Waters X-Terra Shield, RP18 50 x 4.6 mm, 3.5 μm and flow rate increased to 1.5 mL/ min and the system changed to gradient program as shown in Table 1), good resolution and separation of peak was observed as shown in Fig 2(b).

**Table 1:** Gradient program for the mobile phase during analytical method development.

Time (min)	Mobile phase-A (%v/v) Phosphate buffer pH 3.5	Mobile phase-B (%v/v) Acetonitrile
0	95	5
1	95	5
5	20	80
6	20	80
6.1	95	5
8	95	5

To confirm the assumption that the peak which eluted at about 1.5 min is of fumarate, fumaric acid was injected at same chromatographic conditions and similar peak was observed.

It can be observed that with the optimized chromatographic conditions shown in Table 2, the retention time was significantly reduced to 5.941 min in comparison to reported retention times in literature [11, 5, 6]. This can reduce the run time and the cost of analysis of samples. All the reagents used are also commonly available.

**Table 2:** Optimized chromatographic conditions.

Equipment	HPLC with UV Detector—Waters (Alliance)
Mobile phase	Mobile phase A: Phosphate buffer pH 3.5
	Mobile phase B: Acetonitrile
Column	Waters X-Terra Shield, RP18 50x4.6 mm, 3.5 μm
Column temperature	Ambient
Sample cooler	5°C
Injection volume	20μL
Flow rate	1.5 mL/min
Wavelength	260 nm
Diluent	Water:Methanol (50:50%v/v)

While the composition of mobile phase was similar consisting of phosphate buffer and acetonitrile in various reported methods^11 12^, the retention time of EFV was observed to be drastically reduced in comparison to the reported values (from 13.2 min to 5.951 min) [11]. This may be due to the use of column of different dimension 50 x 4.6 mm, 3.5 μm instead of 250 × 4.6 mm, 5 μm. With the use of the analytical column 150 x 4.6 mm, 3 μm, the retention time of 6.9 min [12] and with 75 x 4.6 mm, 3.5 μm, retention time of 6.45 min have been reported [6]. While the pH of the mobile phase reported are higher than 3.5 [11, 12], peak was observed to be split at higher pH in the present investigation. Best results were obtained at a pH of 3.5. Most of the investigations reported in literature have used flow rate of 1 mL/min [11, 12] or less [6, 13], in the present investigation TDF peak was observed to be very close to the void volume at flow rate of 1 mL/min. Proper retention time for EFV and TDF were observed at 1.5 mL/min. Higher flow rate would have also contributed for lowering the retention time of EFV. The lower retention time can help to reduce the time required for analysis. With the use of the gradient elution method, improved resolution, increased detection, shorter analysis time and increased efficiency was observed.

### Analytical method validation

Various parameters such as linearity and range, precision, accuracy, sensitivity (limit of detection, limit of quantification), specificity, robustness/ruggedness, stability, were evaluated for validating the developed analytical method as per ICH guidelines [10].

#### Linearity and range

**Fig 3:**
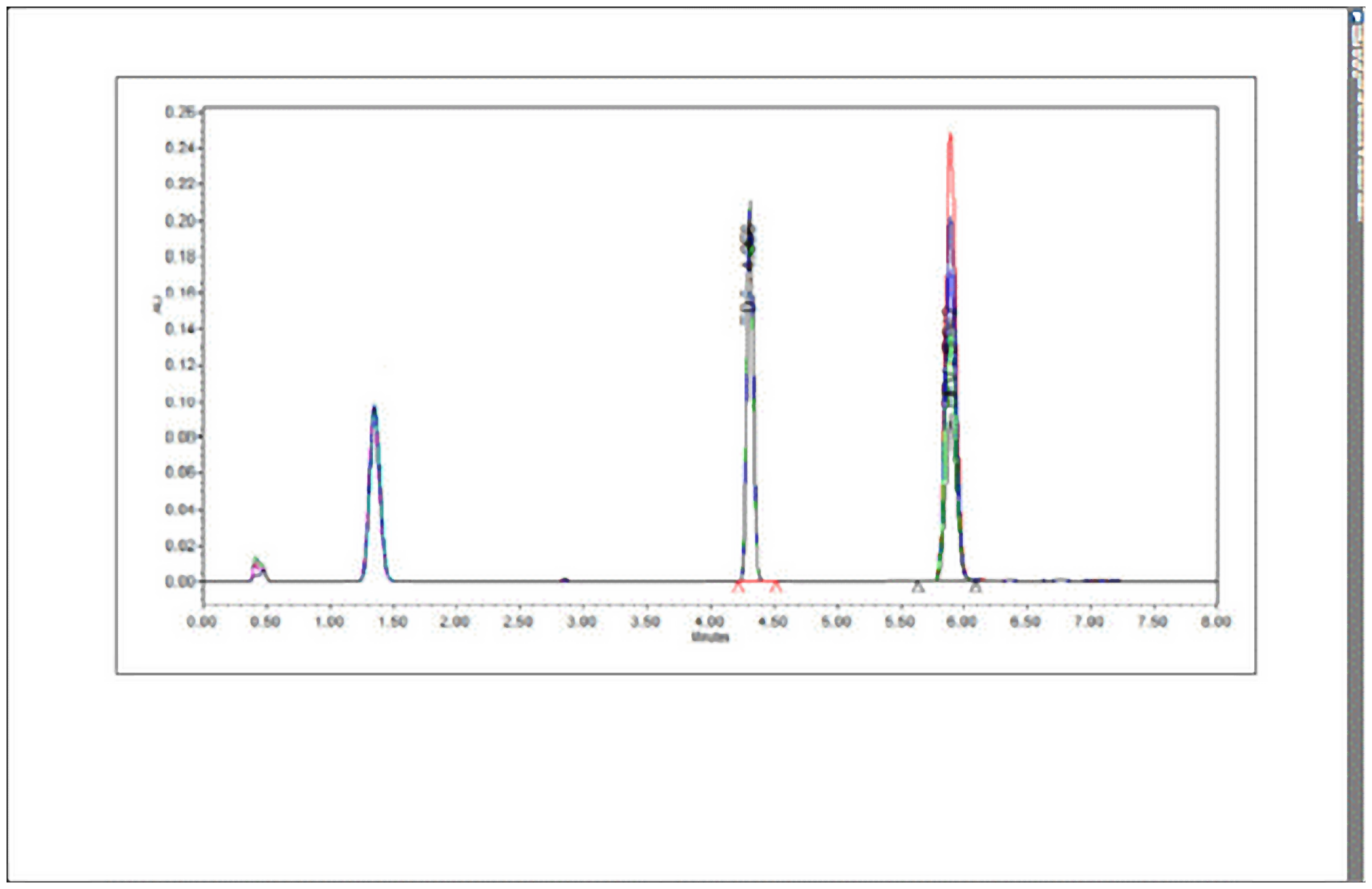
Chromatogram for linearity study.

The linearity of an analytical procedure is its ability (within a given range) to obtain test results which are directly proportional to the concentration (amount) of analyte in the sample. The chromatograms (overlay plot) obtained for the linearity study is shown in Fig 3. Calibration curve was constructed by plotting the peak area ratios (analyte/internal standard) v/s concentration. Regression equation obtained was as given in Eq 1.

y=0.0183x+0.0403 (1)

The calibration plot revealed linearity in the concentration range of 1–300 μg/mL with the correlation coefficient of 0.9996.

As per the extensive literature review, most of the methods were reported to give linear relation in the concentration range upto 10 μg/mL [6, 7, 12, 14]. Only a few had reported linearity at higher concentration of 0.1–100 μg/mL [5]. In one of the reported methods for simultaneous estimation of Emtricitabine, Tenofovir and EFV, the linearity was investigated in the concentration range of 200–280 μg/mL [15]. The present investigation assures the linearity in a wider range of 1–300 μg/mL.

#### Precision

**Fig 4:**
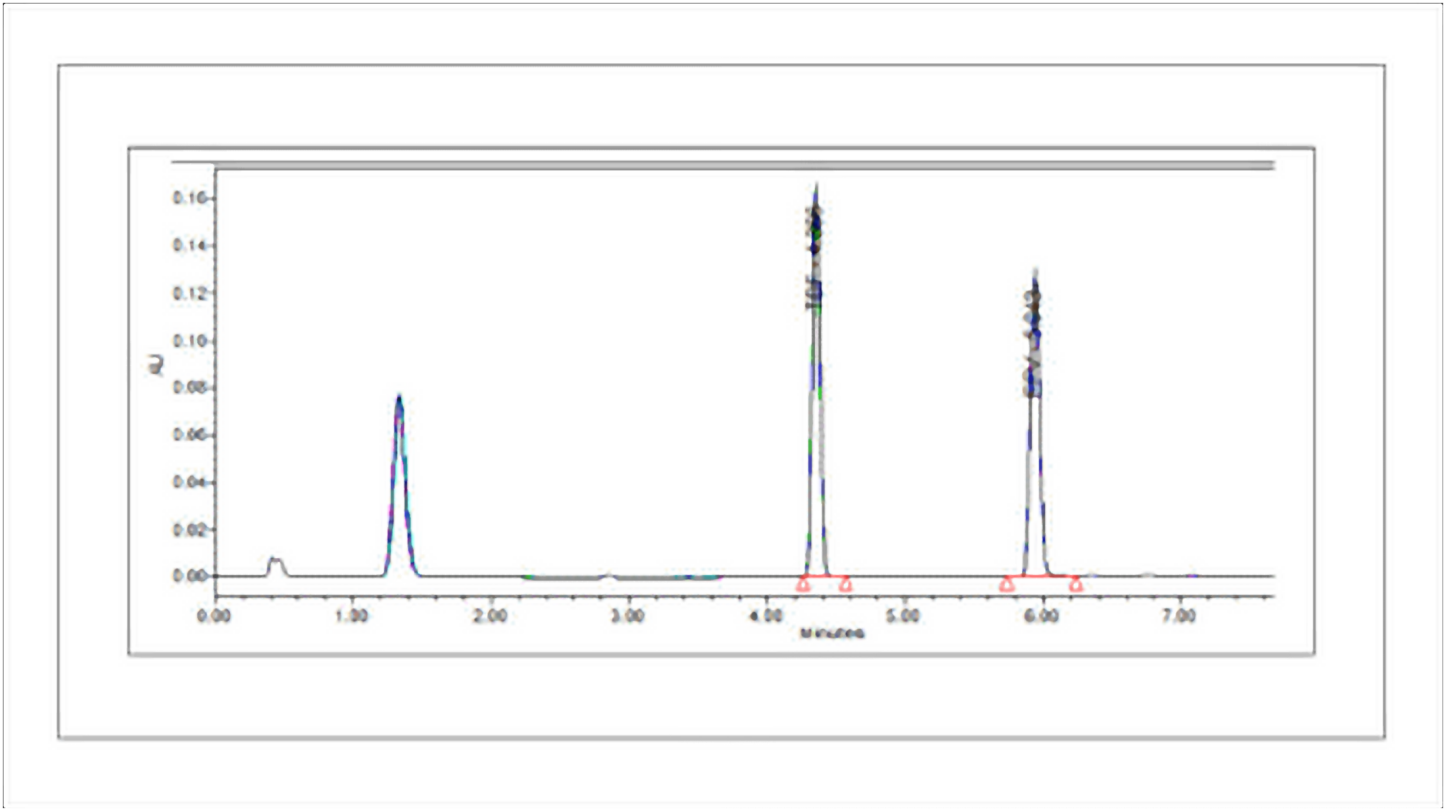
Chromatogram for precision study.

The precision of an analytical procedure expresses the closeness of agreement (degree of scatter) between a series of measurements obtained from multiple sampling of the same homogeneous sample under the prescribed conditions. The precision (repeatability) of the analytical method was determined by assaying six samples at 100% test concentration. RSD was determined to be 0.497 (NMT 2.0%). The chromatograms obtained for the precision study is shown in Fig 4.

The intra-day and inter-day precisions were also assessed by analyzing three samples at three different times of the same day and on three consecutive days respectively and RSD were found to be less than 1.0% which is within the normal acceptable range (NMT 2.0%). RSD of upto 8.6% have been reported in the literature for precision of the analytical methods for estimation of EFV [12,14]. Although many authors have not analyzed the data statistically with ANOVA [12, 4–7], a few have. [13,14] In the present investigations, no significant differences were observed for results obtained for intra-day and inter-day data when statistically analyzed with ANOVA (p>0.05).

#### Accuracy

The accuracy of an analytical procedure expresses the closeness of agreement between the value which is accepted either as a conventional true value or an accepted reference value and the value found. The accuracy of HPLC method was determined by recovery study. Six replicates were analyzed at 100% concentration level and three replicates each at 50% and 150% concentrations. Assessment of accuracy was accomplished by evaluating the percent recovery of the analyte as shown in Table 3.

**Table 3:** Data of recovery study for accuracy parameter.

Sample details	Area of EFV*	Area of TDF*	Ratio*	Amount found* (μg/mL)	Amount added* (μg/mL)	% Recovery*
Recovery at 50% level (n = 3)	1844694 ± 7156	7829094 ± 15276	0.235 ± 0.002	12.534 ± 0.076	0.983 ± 0.004	99.933 ± 0.611
Recovery at 100% level (n = 6)	3626186 ± 16030	7845639 ± 31474	0.462 ± 0.002	25.122 ± 0.098	2.005 ± 0.008	100.15± 0.404
Recovery at 150% level (n = 3)	5421860 ± 29968	7842032 ± 43622	0.691 ± 0.003	37.855 ± 0.138	3.02 ± 0.011	100.6 ± 0.361

*Data expressed as Mean ± SD

It can be observed that the percent recovery determined falls in the range of 99 to 101%. As per the literature review, % recovery achieved with several the reported methods are between 97–103% [11, 12] or even wider range[7, 14].

#### Limit of detection (LOD) and limit of quantification (LOQ)

The detection limit of an individual analytical procedure is the lowest amount of analyte in a sample which can be detected but not necessarily quantitated as an exact value. The quantitation limit of an individual analytical procedure is the lowest amount of analyte in a sample which can be quantitatively determined with suitable precision and accuracy. Out of the various methods described, LOD and LOQ were determined based on signal-to-noise ratio. A signal-to-noise ratio between 3 or 2:1 is generally considered acceptable for estimating the detection limit and a typical signal-to-noise ratio for LOQ is 10:1 [10].

Signal for known low concentration (0.03μg/mL) of analyte was compared with blank sample for the LOD. Signal to noise ratio was found to be 7:1 which was in accordance with the acceptance criteria indicating the detection at concentration of even as low as 0.03 μg/mL concentration also with reliability. Signal-to-noise ratio of more than 20:1 were obtained with known low concentrations (six replicates of 0.1 μg/mL solution) of analyte which indicated that even 0.1 μg/mL of EFV may also be reliably quantified. The reported therapeutic range of EFV is 1–4 μg/mL [16]. Hence the developed method has the potential to estimate even the minimum concentration of drug in effective therapeutic range.

The results obtained are similar with the reported LOD and LOQ determined by dispersive liquid-liquid micro-extraction coupled to high performance liquid chromatography (DLLME) with 0.01 and 0.1 μg/mL [5,12], 0.02 and 0.05 μg/mL [7], as LOD and LOQ respectively.

#### Specificity

**Fig 5:**
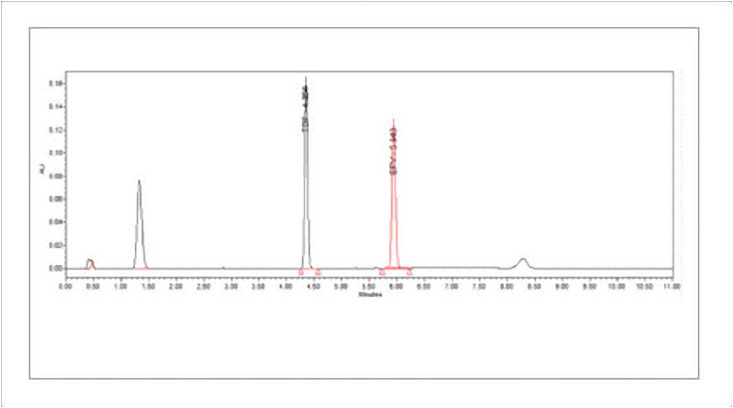
Chromatogram for specificity study.

Specificity is the ability to assess unequivocally the analyte in the presence of components which may be expected to be present. The peak areas of compounds co-eluting with one of the analytes should be less than 20% of the peak areas of the analyte at LOQ. For compounds co-eluting with the internal standard the peak area should be less than 5% of the internal standard area [17]. Specificity of the developed method was checked by recording chromatogram of EFV and TDF in the presence of placebo. No interference was observed at the retention time of EFV and TDF in placebo and diluent (as shown in Fig 5) indicating the specificity of the analytical method.

#### Robustness

The robustness of an analytical procedure is a measure of its capacity to remain unaffected by small, but deliberate variations in method parameters and provides an indication of its reliability during normal usage. The robustness of the method was studied by changing the flow rate (±0.2 mL/min), wavelength (±2 nm) and pH of mobile phase (±0.2). Robustness of the developed method was determined in terms of RSD as shown in Table 4.

**Table 4:** Robustness data for change in different parameters.

Parameters		RSD (%)	
		EFV	TDF
Change in flow rate (±0.2 mL/min)	Retention time	2.288	4.602
	Area	1.573	0.554
	Tailing factor	0	0
	Resolution	4.604	4.604
Change in wavelength (±2 nm)	Retention time	2.391	4.042
	Area	1.009	0.521
	Tailing factor	0	0
	Resolution	3.262	3.262
Change in pH of mobile phase (±0.2)	Retention time	2.391	4.042
	Area	1.009	0.521
	Tailing factor	0	0
	Resolution	3.262	3.262

No significant changes in the chromatographic parameters were observed when changing the optimized experimental conditions (flow rate, wavelength, pH of mobile phase). Robustness of the developed method was determined in terms of RSD as shown in Table 4. The RSD in all the investigations were found to be less than 5. Robustness with respect to change in flow rate, wavelength and pH has not been investigated in various reported results [6, 14, 17]. RSD values of upto 7.5 have been reported [5].

#### Stability of analytical solution

Stability of the analytical solution was tested at 2, 4, 6, 12, 18, 24 hours when stored at ambient temperature, after 10 days when stored at 4°C, and compared with chromatograms of freshly prepared sample. Stability sample results were found to be within 15% of nominal concentrations. No significant change (p > 0.05) was observed indicating the stability of the analytical solution for 10 days, the time being based on the expected duration of analysis. During freeze-thaw stability evaluations, the freezing and thawing of stability samples was found to mimic the intended sample handling conditions to be used during sample analysis. Stability of the samples upto 6 hours at room temperature has been reported in other similar investigations [6]. The stability of EFV in human plasma when stored at −20°C has been reported for upto 30 days [7].

## Conclusion

A simple and sensitive reversed-phase HPLC gradient method has been developed and validated for the estimation of EFV in plasma using UV detector. A good resolution was obtained between EFV and TDF as internal standard with retention time 5.941 minutes and 4.356 min respectively. No interference peaks were observed around the retention time of EFV and TDF. The method was found to be linear (R^2^ = 0.9996) within the analytical range of 1–300 μg/mL. The results obtained suggested that the method was accurate and reproducible and the drug was stable in plasma. Therefore, the developed chromatographic method can be used for estimation of EFV in plasma with good resolution to evaluate the pharmacokinetic parameters of EFV. The developed method has also been used successfully in a recently published investigation for the estimation of EFV in plasma after its intranasal administration [18].
